# Tonsillotomy

**Published:** 1883-05

**Authors:** Charles C. F. Gay

**Affiliations:** Surgeon to the Buffalo General Hospital


### Tonsillotomy.

By Charles C. F. Gay, M. D.,

Surgeon to the Buffalo General Hospital.

This is an operation so often neglected, to the detriment of those afflicted with tonsillar disease and tonsillar enlargement, that I deem the subject of sufficient importance again to call attention to it. The necessity for the performance of this operation is apparent to any one accustomed to frequent inspection of the throats of patients. The chronically diseased and enlarged tonsil acts as a foreign substance in the throat; it causes irritation and excites coughing; the tonsil becomes more inflamed whenever a cold is contracted, and, unless timely removed, it leads the way to the advent of phthisis, and is, indeed, a predisposing cause of this affection. The gland, on account of chronic disease, may have ceased to perform the function of a gland; physiologically, it is no longer a secreting organ; hence, the last and only objection to its ablation is disposed of and there no longer exists reason for its preservation.

As an objection to excision I ought not to omit to state that impairment of the virility of the individual is adduced as an argument against tonsillotomy; but the weight of authority is opposed to this theory. More recent writers have been able to adduce abundant evidence to disprove the doctrine just alluded to. That the procreative power should have been believed to be at all affected by removal of the tonsils, is as strange as it is absurd. The adoption of such a theory may be regarded as another instance of the wonderful facility of the medical mind for embracing medical vagaries. I herewith report, in brief, a few illustrative cases, notes of which were taken immediately or soon after the performance of the operation.

*Case* 1. J. H., female; aged 16 years; has been troubled for years with tonsillitis. She has a cough and her general health is impaired; the left tonsil is much enlarged and indurated; it is about the size and shape of a small butternut. I excised the tonsil, using the tonsillotome. For a moment there was considerable hemorrhage, which ceased spontaneously. Patient had no subsequent trouble; cough disappeared; health was restored and remained good thereafter.

*Case* 2. J. B., aged 4 years; male; has had much trouble with his throat; frequent attacks of double tonsillitis. The tonsils have, in consequence, become much hypertrophied and affect the voice and impair the speech. He was put under ether and the entire right and one-half of the left tonsil excised with the tonsillotome. But little hemorrhage followed; recovery was uninterrupted, and thereafter health was good, with no return of the tonsillar enlargement.

*Case* 3. N. T., female; aged 8 years; has enlarged tonsils, which are always troublesome and get inflamed whenever she contracts a cold. Put under ether and the right tonsil removed. But little hemorrhage followed the operation. Excepting some soreness of the throat, there was no complaint during the after-treatment.

*Case* 4. Child, aged 7 years; has enlargement of both tonsils. They are troublesome in speaking and sleeping. Excised the left tonsil, it being the larger of the two, and left the other with the hope that removal of the one would cause absorption of the other. Hemorrhage arrested by ferri persulphatis solution. Patient passed from subsequent observation.

*Case* 5. Child, aged 17 months; has had enlarged tonsils, the mother says, several months; has difficulty in sleeping; snores and is wakeful. Both tonsils are enlarged. Ether was given when I excised the right tonsil. As no diminution in size of the other followed, it also was removed seven months afterwards. Since recovery nothing has been heard of the child.

*Case* 6. G. S., male; aged 6 years. Tonsils both very large and the child cannot sleep soundly in consequence. Excised right tonsil under ether. Child made a good recovery; the left tonsil became so much absorbed as to give no trouble.

*Case* 7. Miss C., aged 23 years; became alarmed on account of a chronic cough; both of her tonsils are chronically enlarged; right tonsil is the larger of the two; they meet; obstruct respiration; her voice is affected and swallowing is difficult. She is worse whenever she contracts a cold. I removed the right tonsil with the tonsillotome without ether, cutting it close at its base. Hemorrhage was profuse, and was finally arrested by a sponge probang, saturated with vinegar, held firmly for two or three minutes upon the wound. Sol. of persulph. ferri and ice had been first employed without effect. There was no secondary hemorrhage. Process of granulation confined her to the house for one week. Cough and general health are much improved.

*Case* 8. Miss F., aged 20 years; has enlargement of both tonsils. She says she had acute tonsillitis three months ago.

She was etherized. I excised the right tonsil. One month later she was again etherized and the left tonsil was removed. This operation was followed by alarming hemorrhage, which, however, was arrested, after failure from the application of ice and sol. ferri persulphatis, by creosote, applied by a sponge probang. In five days the patient was entirely recovered.

COMMENTS.—One may have reasonable expectation of the absorption of the remaining tonsil after complete removal of the larger one, but this result cannot always be depended upon; it is advisable, however, to wait a reasonable length of time for the process of absorption to accomplish what we wish, unless the size of the remaining tonsil be such as to make the propriety of its immediate removal unquestionable.

I have never known permanent advantage to follow partial ablation of a tonsil; excision, entire and complete, should always be practiced. Ordinarily, there will be little or no permanent diminution in the size of the gland after its partial or incomplete removal. There will likewise be greater probability of dangerous hemorrhage by partial than by complete excision.

Operators have their preferences in the choice of instruments. My own preference is the tonsillotome in all cases, and I prefer the instrument that cuts from within out. My own instrument is a modification of Fahnestock's, manufactured by Charrier, Paris. It is furnished with a fork with barbed points. I have modified this instrument slightly by removing the beard from one of the prongs of the fork, so as to enable me to disengage more easily the tonsil from the fork after excision.

In operating I always prefer that the patient be etherized and to have the tongue depressed by an assistant.

Standing in front of my patient, I hold the instrument in my right hand in excising the left tonsil, and use the left hand to hold the instrument in excising the right tonsil. Placing the instrument over the tonsil, firmly holding it at the base of the gland, the tonsil is transfixed by the fork and drawn through the fenestrum. The more the tonsil is put upon the stretch and drawn tensely by the fork, the easier and more surely will excision be accomplished.

It is by no means always necessary to etherize an adult patient or employ an assistant to depress the tongue. Rigid rules in this regard—as in most instances in surgical practice—cannot be prescribed and carried out. There are patients who—on account, perhaps, of possessing less irritable throats than others, or who may have been accustomed to local applications—have almost complete control over their muscles, in which case the surgeon will have no difficulty in operating without ether or an assistant.

